# Altered Processing of Amyloid Precursor Protein in Cells Undergoing Apoptosis

**DOI:** 10.1371/journal.pone.0057979

**Published:** 2013-02-28

**Authors:** Tina Fiorelli, Lisa Kirouac, Jaya Padmanabhan

**Affiliations:** 1 USF Health Byrd Alzheimer's Institute, University of South Florida, Tampa, Florida, United States of America; 2 Department of Molecular Medicine, University of South Florida, Tampa, Florida, United States of America; University of Pittsburgh, United States of America

## Abstract

Altered proteolysis of amyloid precursor protein is an important determinant of pathology development in Alzheimer's disease. Here, we describe the detection of two novel fragments of amyloid precursor protein in H4 neuroglioma cells undergoing apoptosis. Immunoreactivity of these 25–35 kDa fragments to two different amyloid precursor protein antibodies suggests that they contain the amyloid-β region and an epitope near the C-terminus of amyloid precursor protein. Generation of these fragments is associated with cleavage of caspase-3 and caspase-7, suggesting activation of these caspases. Studies in neurons undergoing DNA damage-induced apoptosis also showed similar results. Inclusion of caspase inhibitors prevented the generation of these novel fragments, suggesting that they are generated by a caspase-dependent mechanism. Molecular weight prediction and immunoreactivity of the fragments generated suggested that such fragments could not be generated by cleavage at any previously identified caspase, secretase, or calpain site on amyloid precursor protein. Bioinformatic analysis of the amino acid sequence of amyloid precursor protein revealed that fragments fitting the observed size and immunoreactivity could be generated by either cleavage at a novel, hitherto unidentified, caspase site or at a previously identified matrix metalloproteinase site in the extracellular domain. Proteolytic cleavage at any of these sites leads to a decrease in the generation of α-secretase cleaved secreted APP, which has both anti-apoptotic and neuroprotective properties, and thus may contribute to neurodegeneration in Alzheimer's disease.

## Introduction

Altered proteolysis of the amyloid precursor protein (APP) is a central event in the development of pathology associated with Alzheimer's disease (AD). Cleavage of APP by β- and γ-secretases produces amyloid-β (Aβ), the primary component of amyloid plaques [1], [2]. β-secretase cleaves APP between amino acids 671 and 672, while γ-secretase cleaves in the vicinity of amino acids 711–714 (based on APP_770_). APP is also cleaved by α-secretases within the Aβ region, precluding the formation of Aβ [3], [4]. In addition, other proteases such as calpains and caspases are also known to cleave APP [5]–[10]. For example, in apoptotic cells APP is cleaved by caspase-3 at three distinct sites [6], [11]. Two of these sites, DNVD*S_198_ and DYAD*G_220_, are located near the N-terminus of APP, while a third site, VEVD*A_740_, is located near the C-terminus. Since the cleavage at VEVD*A_740_ could be prevented with the small peptide inhibitor DEVD-FMK, this cleavage has been attributed to caspase-3 [6].

Caspase activation, DNA fragmentation, and apoptosis are all associated with neurodegeneration in AD brains [5], [12]–[15]. Studies in post mortem AD brain tissue have shown increased levels of caspases, specifically caspase-1 and -7, prior to exhibition of other signs of apoptosis [16]. Induction of caspases early in AD pathogenesis, along with the observation that caspases can cleave APP, suggests that caspase-mediated processing of APP may contribute to pathology development in AD [5]. Caspases-3, -6, and -8 have all been implicated in APP cleavage [6], [17]. Here, we examined caspase-dependent processing of APP under apoptotic conditions, and present evidence for the generation of two novel proteolytic fragments between approximately 25 and 35 kDa in size and immunoreactive to antibodies against the Aβ-region and the C-terminal caspase site. *In vitro* studies show the formation of one of these fragments in primary neurons, and both of these fragments in H4 neuroglioma cells undergoing apoptosis. Our studies using the caspase inhibitors Z-DEVD-FMK and Z-VAD-FMK, and shRNA to caspase-3 and caspase-7, show that the cleavage of APP during apoptosis is more specific to caspase-7 than caspase-3. The production of these specific proteolytic fragments, as well as the involvement of caspase-7 in the cleavage of APP, suggests a potentially pathogenic role for this caspase in APP processing and neurodegeneration and warrant further investigation.

## Materials and Methods

### Ethics statement

Studies involving animals (primary neuronal culture) were done in accordance with the rules and regulations set forth by the University of South Florida's Institutional Animal Care and Use Committee (IACUC). This specific study was approved by the University's IACUC committee (Protocol # R3758). Timed pregnant rats at E14 were purchased from Harlan and cared for by the well-established animal care facility at University of South Florida (USF), which is accredited by the American Association of Laboratory Animal Care (AALAC).

### Cell culture and drug treatment

Cortical neurons were cultured following published protocols [18]. Briefly, timed pregnant rats were anesthetized with Somnasol solution and E18 embryos were collected. Embryonic brains were removed and triturated in 0.25% trypsin. Dissociated cortices were centrifuged and cells resuspended in neurobasal media with B27 supplement and plated onto poly-L-lysine coated culture plates at a density of approximately 200,000 cells/cm^2^. Cells were cultured for seven days prior to initiation of experiments. Apoptosis in neurons was induced by treatment with 10 µM camptothecin (CPT) [19]–[21]. The neurons were treated with CPT in the presence or absence of the group II caspase inhibitor Z-DEVD-FMK or the broad-spectrum caspase inhibitor Z-VAD-FMK (R&D Systems, Minneapolis, MN) at 10–20 µM concentrations. Neurons were incubated for one hour with inhibitors and 12 hours with CPT prior to collecting samples for western blot analysis [18], [22].

H4 cells expressing APP (H4-APP) were a kind gift from Dr. Todd Golde (Mayo Clinic, Jacksonville, FL). The generation of this cell line has been previously described [23]. These cells were cultured in Opti-MEM (Life Technologies) media containing 10% FBS (Atlanta Biologicals, Lawrenceville, GA), 1% penicillin/streptomycin (Life Technologies), and 50 µg/ml hygromycin (normal growth media) until they reached 60% confluence. For experiments involving serum deprivation, cells were rinsed twice with DPBS and cultured in DMEM (Life Technologies) containing penicillin/streptomycin with no serum (serum deprivation media) for 48 hours. After 48 hours, cells were rinsed with DPBS twice more before adding the appropriate treatment media. For experiments involving caspase inhibition, cells were cultured in 10–20 µM Z-DEVD-FMK or Z-VAD-FMK in normal growth media for one hour prior to induction of apoptosis. Cells were then exposed to 10 µM CPT for indicated time periods in the presence and absence of inhibitors.

### Lysate preparation

After treatment for the indicated times, cells were scraped into culture media and pelleted by centrifugation at 500×g for five minutes at 4°C. Pellets were washed three times in ice cold PBS and resuspended in ice cold RIPA lysis buffer containing protease inhibitors (1 mM PMSF, 1 mM NaVO_4_, 1 mM NaF, and one Complete Mini protease inhibitor tablet per 10 mL (Roche Applied Science, Indianapolis, IN). Cells were lysed by sonication using a Model 100 Sonic Dismembrator (Thermo Fisher Scientific) on setting six for ten seconds on ice and the insoluble fraction separated by high-speed centrifugation (20800×g for 15 min at 4°C). The supernatant containing soluble proteins was retained and analyzed for total protein content using a Bradford or bicinchoninic acid (BCA) assay (Thermo Fisher Scientific, Rockford, IL) following the manufacturer's protocol. Samples containing equal amounts of protein were prepared in tricine or Laemmli sample buffer for polyacrylamide gel electrophoresis (PAGE) and western blot analysis.

### Western blot analysis

Western blot analyses were performed following established protocols [18], [22]. Briefly, samples were boiled with tricine or Laemmli samples buffer and separated by PAGE on either 10–20% tricine (Life Technologies, Grand Island, NY) or 15% tris-glycine mini gels, respectively, at 90–125 V. Molecular weight markers used were either SeeBlue Plus2 (Invitrogen) or Precision Plus Dual Xtra (Bio-Rad). Proteins were transferred to 0.1 or 0.2 µm nitrocellulose membranes using the XCell SureLock mini-blotting module (Invitrogen) at 30 V for two hours. Membranes used for detection of Aβ were boiled in PBS for five minutes prior to blocking. Membranes were blocked in 5% non-fat dry milk in TBS for one hour prior to probing with primary antibodies overnight. Primary antibodies diluted in 3% bovine serum albumin (BSA) included p53, caspase-3, caspase-7, cleaved caspase-3, cleaved caspase-7, poly ADP-ribose polymerase (PARP) (Cell Signaling, Beverly, MA), 6E10 (Covance, Princeton, NJ), caspase-cleaved APP (Millipore, Billerica, MA), and β-actin (Sigma, St. Louis, MO). Blots were washed four times in PBS containing 0.05% Tween-20 and probed with appropriate secondary antibodies, either goat anti-mouse or donkey anti-rabbit IgG conjugated to horseradish peroxidase (Thermo Fisher Scientific), for two hours at room temperature. After further washes, blots were developed using Super Signal West Pico Enhanced Chemiluminescence (ECL) Substrate (Thermo Fisher Scientific). Images captured on autoradiography film (MidSci, St. Louis, MO) were scanned for digitization using a flatbed scanner, and densitometric analysis was performed using ImageJ image processing and analysis tool.

### Immunoprecipitation

Equal number of cells were plated onto 100 mm tissue culture dishes and cultured for 24 hours, after which point cells were treated with fresh normal growth medium in the absence or presence of CPT (10 µM) for different time periods. At each time point, tissue culture supernatant from each culture dish was collected and spun down at 1,000 rpm for five minutes to remove cell debris. The supernatants were subjected to immunoprecipitation analysis for secreted APP and Aβ following established protocols [22]. Briefly, the tissue culture supernatants were incubated with 2 µl of 6E10 antibody for two hours at 4°C by end-over-end rotation. Thirty μl (packed volume) of protein G sepharose beads were added, and incubation continued overnight. A sample incubated with no primary antibody and only protein G sepharose beads served as control for the immunoprecipitation experiments. At the end of the incubation beads were collected by centrifugation at 3,300×g for 30 seconds and washed with RIPA lysis buffer four times. The pelleted beads were boiled with Laemmli sample buffer to solubilize the antigen-antibody complex, separated on a 10–20% tris-glycine gel, and western blot analysis performed as described above using 6E10 antibody to determine secreted levels of APP and Aβ.

### Immunostaining analyses

Cells were plated in eight-chamber slides (Nunc, Rochester, NY) coated with poly-L-lysine (Sigma). After the indicated treatments, cells were fixed with 4% para-formaldehyde and immunostaining analysis was performed following established protocols [18], [22], [24]. Slides were washed with PBS and blocked with 1% BSA in TBS containing 0.1% Triton X-100 for one hour. Slides were then incubated overnight at 4°C in primary antibodies diluted in blocking buffer. These included 6E10 (1∶500), cleaved caspase-3 (1∶250), cleaved caspase-7 (1∶250), and MAP2 (1∶1000). Following incubation, slides were washed four times with PBS for five minutes per wash and incubated with secondary antibodies (goat anti-mouse AlexaFluor 488 (1∶1000) and goat anti-Rabbit AlexaFluor 594 (1∶4000)) for two hours in the dark. Slides were then washed twice, counterstained with 1 µg/mL Hoechst stain diluted in PBS for five minutes, and washed thrice more before coverslipping using Fluorogel (Electron Microscopy Sciences). Stained cells were visualized using a Zeiss Imager.Z1 microscope and analyzed using AxioVision Rel 4.6.3 software.

### Flow cytometry

Cells were lifted using 0.05% trypsin/EDTA for two to three minutes, followed by neutralization of trypsin using normal growth media. Cells were pelleted by centrifugation at 500×g for five minutes at 4°C and pellets washed with PBS twice, centrifuging at 500×g for five minutes at 4°C between washes. Pelleted cells were resuspended in one part PBS and fixed by adding nine parts ice cold 70% ethanol, added drop-wise while vortexing at a low speed. Fixed cells were stored at −20°C for no more than one week before analysis. Fixed cells were pelleted by centrifugation at 200×g for five minutes and washed twice with PBS. Cells were then resuspended in staining solution containing 5 µg/mL propidium iodide, 0.1% Triton X-100, and RNaseA in TBS and incubated at room temperature for 45 minutes. Samples characterizing apoptosis in H4-APP cells were then analyzed using a BD LSR II flow cytometer. Results were modeled for cell cycle progression and apoptosis using ModFit LT. Samples involving caspase inhibitors were analyzed using an AccuriC6 cytometer with CFlow Plus analysis software.

### shRNA knockdown of caspases

Four different shRNA clones to both human caspase-3 and caspase-7 in pGFP-V-RS vector (TG305638) were obtained from Origene Technologies (Rockville, MD) and transfected into H4-APP cells using Turbofectin 8.0 transfection reagent (Origene Technologies, Rockville, MD) following the manufacturer's protocol. Since these plasmids allow expression of GFP by a CMV promoter, the transfection efficiency could be verified by analyzing the cells for GFP expression using a Zeiss Observer.A1 inverted microscope with fluorescence capabilities. Down-regulation of the corresponding caspases was verified by western blot analysis using caspase-3 and caspase-7 antibodies prior to using these cells for CPT treatment. An empty vector and a scrambled non-effective shRNA-containing vector were used as controls in transfection studies.

### Statistical analyses

Experiments involving time courses with CPT were analyzed using a two-way ANOVA (time and treatment). shRNA and inhibitor effects were analyzed with a one-way ANOVA when CPT was not present and a two-way ANOVA in the presence of CPT. All post-hoc analyses were done using the Tukey-Kramer method. All statistical analyses were performed using NCSS-PASS.

## Results

### DNA damaging agents induce apoptosis in H4-APP neuroglioma cells

**Figure 1:**
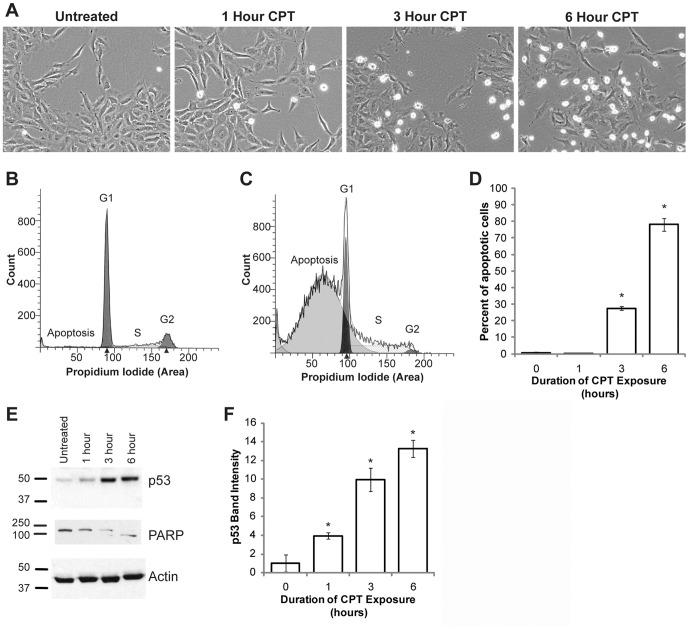
CPT induces apoptosis in H4-APP neuroglioma cells. (A) H4-APP cells were exposed to 10 µM CPT for zero to six hours and examined using brightfield microscopy. Morphological changes indicative of apoptosis, including cell detachment and shrinkage, were noted after three hours of treatment and were most pronounced at six hours. (B) ModFit LT modeling of flow cytometry data obtained from untreated H4-APP cells stained with propidium iodide. In untreated cells, no sub-G1 population is observed. (C) Flow cytometric analysis of cells treated with 10 µM CPT for six hours and stained with propidium iodide showed a significant increase in sub-G1 cells, indicative of apoptosis. (D) Histogram of flow cytometry data from all time points across two independent experiments. A significant increase in apoptotic cells was identified at three and six hours in cells treated with CPT. Asterisks indicate significant difference from all other groups, p<0.001. (E) Representative blot from three independent experiments performed to determine the levels of p53 and PARP. CPT-induced apoptosis was associated with a time-dependent induction in p53 (E, top panel) and cleavage of PARP (E, middle panel). Reprobe of p53 blot with an antibody against β-actin showed equal protein loading (E, bottom panel). (F) Quantification of p53 blot showed a significant induction after one hour, p<0.001. Post hoc analysis revealed that each group is significantly different from all other groups.

In order to examine the proteolytic processing of APP during apoptosis, we treated H4 neuroglioma cells expressing human APP (H4-APP cells) with the DNA damaging agent camptothecin (CPT), a topoisomerase inhibitor [25], [26]. Cells were synchronized by serum starvation for 48 hours, then serum stimulated in the presence of 10 µM CPT for zero to six hours. Apoptosis induction was assessed by flow cytometry, light microscopy, and western blot analyses. Cellular shrinkage, nuclear condensation, and detachment, characteristics of cells undergoing apoptosis, were observed after three hours of CPT treatment and were more pronounced after six hours (Figure 1A). Flow cytometric analysis of propidium iodide stained cells revealed a significant increase in the number of cells with sub-G1 DNA content, indicative of apoptosis (Figure 1B and 1C, control and CPT treated respectively). Quantification indicated that CPT treatment induced apoptosis in approximately 78% of cells after six hours (Figure 1D). Since DNA damage induced apoptosis has been associated with p53-dependent mechanisms [27]–[29], we examined whether the CPT-mediated cell death in H4 cells was associated with an induction of p53. Western blot analysis of the lysates from H4-APP cells treated with CPT showed a time-dependent increase in p53 levels, suggesting that the cell death we observed is p53-dependent (Figure 1E, top panel). Next, we examined the levels of poly-ADP ribose polymerase (PARP), an enzyme involved in several cellular functions including DNA repair, cell cycle progression, and cell death [30], [31], in cells treated with CPT. PARP is an approximately 116 kDa protein which, under apoptotic conditions, gets cleaved by caspases to generate fragments of approximately 89 kDa and 24 kDa [32]. Analysis of the cell lysates from H4-APP cells treated with CPT showed that the levels of full length PARP were reduced over the time course. This was associated with the appearance of a lower band of ∼89 kDa, indicating caspase-mediated PARP cleavage was associated with the induction of apoptosis upon CPT treatment (Figure 1E middle panel). Normalization for protein loading was done using an antibody against β-actin (Figure 1E, bottom panel). Figure 1F shows the quantification of p53 levels in CPT treated cells from three independent experiments, with asterisks indicating a significant difference from all other groups, p<0.05. These data demonstrate that CPT induces significant apoptosis in H4-APP cells within six hours of treatment.

### Generation of novel APP proteolytic fragments in cells undergoing apoptosis

**Figure 2:**
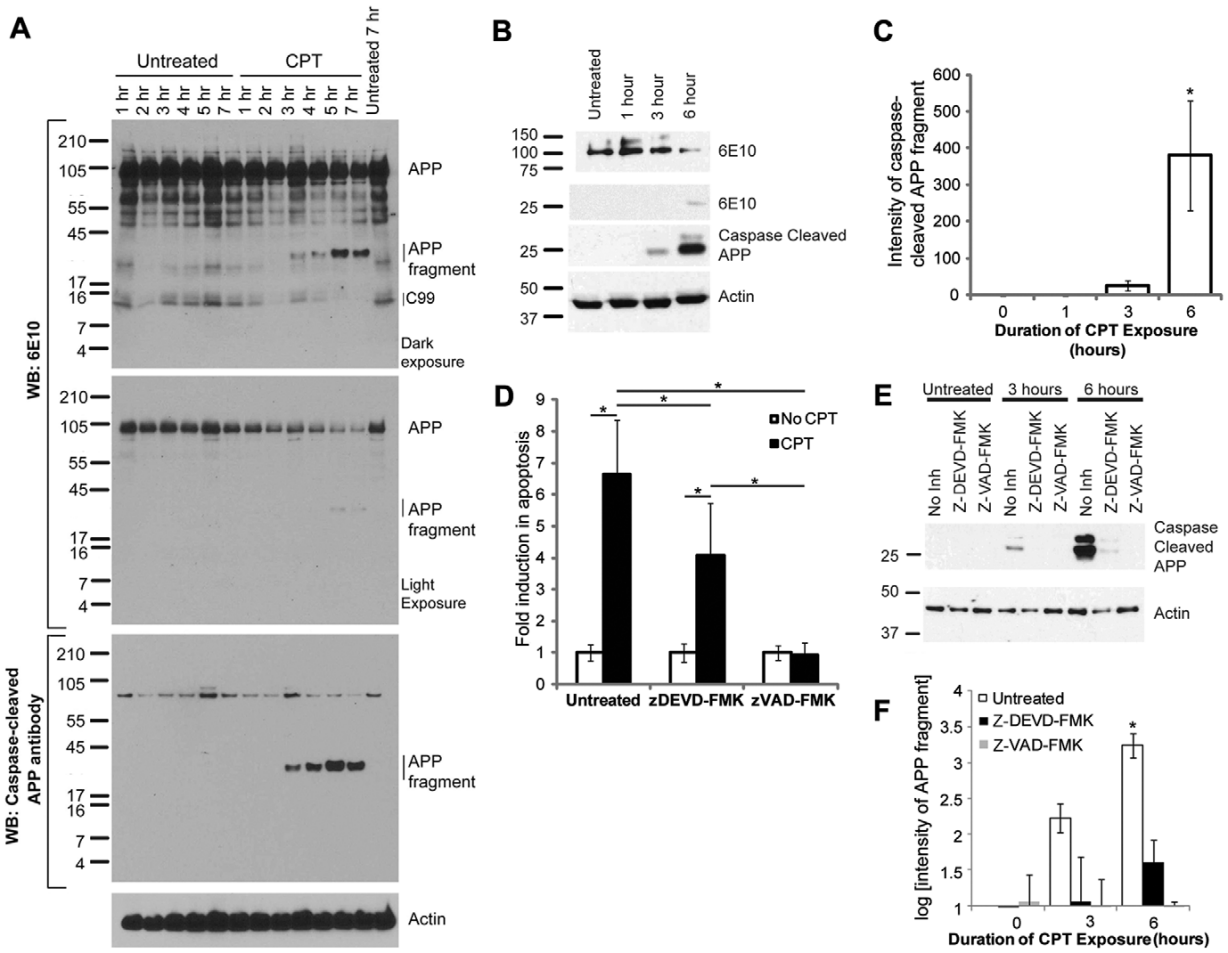
CPT induced apoptosis of H4-APP cells is associated with the formation of novel APP fragments. (A) Lysates from H4-APP cells were separated on 10–20% tricine gel and western blot analysis was performed using 6E10 antibodies. The blot shows a time-dependent increase in the level of APP fragments in apoptotic cells (top panel labeled APP fragments). A short exposure of the blot shows that levels of full length APP are decreased in a time-dependent manner in CPT treated cells (second panel). The blot was re-probed with caspase-cleaved APP antibody, which shows a time-dependent increase in generation of the cleaved fragments in apoptotic cells. The lower panel shows the reprobe of the blot with an antibody against β-actin performed to show protein loading. (B) Lysates from H4-APP cells treated with CPT for one, three, and six hours were separated on a 15% tris-glycine gel and analyzed by western blot using 6E10 (top and second panel) as well as caspase-cleaved APP antibodies (third panel). 6E10 antibody shows the appearance of the cleaved fragment of APP (second panel) with a concomitant decrease in the levels of full length APP (top panel) after CPT treatment. Caspase-cleaved APP antibody shows a much stronger immunoreactivity to the proteolytic fragments (third panel). Probing for β-actin (lower panel) showed equal amount of protein loading on the gels. (C) Quantification of the bottom band detected by caspase-cleaved APP antibody, with normalization to actin, revealed a significant induction in formation of this band after six hours of CPT treatment, p = 0.018. (D) Quantification of the number of cells undergoing apoptosis in the presence and absence of caspase inhibitors and CPT, as analyzed by flow cytometry with propidium iodide staining. Asterisks indicate a significant difference between groups, p<0.05. (E) Western blot analysis of the H4-APP cells showed a strong reduction in the formation of the new fragments in the presence of Z-DEVD-FMK. In the presence of Z-VAD-FMK, the formation of these bands was completely abolished. Reprobe of these blots with an antibody against β-actin shows protein loading on gels. (F) Quantification of the bottom band detected by the caspase-cleaved APP antibody, normalized to actin, from three independent experiments, reveals significant attenuation in the formation of the fragments by caspase inhibitors, with the six hour CPT treated sample showing a significant difference from all other groups, p<0.05. Note that a logarithmic scale is used in this panel.

To assess the proteolytic processing of APP, we performed western blot analyses of lysates from H4-APP cells treated with CPT for different time periods using 6E10 and caspase-cleaved APP antibodies. The 6E10 antibody was raised against amino acids 1–16 of the Aβ region in APP (Covance, Princeton, NJ), while caspase-cleaved APP antibody was raised using a synthetic peptide preceding the aspartic acid residue (APP_739_) in the C-terminal caspase-cleavage site of APP, and affinity purified to specifically detect APP cleaved at this site (Millipore) [6]. Western blot analysis of samples run on 10–20% tricine gels using the 6E10 antibody showed the appearance of a cleaved APP fragment in lysates from cells undergoing apoptosis, but not in those from untreated samples (Figure 2A, top panel). CPT treatment was associated with a decrease in full length APP (Figure 2A top panel and second panel showing a lighter exposure of the full length APP). Together, these results suggest that the 6E10-binding domain of APP is cleaved from the full-length protein during apoptosis. The blots also showed a CPT-dependent decrease in 6E10 reactive APP fragment migrating approximately at 12 kDa, which we believe corresponds to the β-secretase cleaved C-terminal fragment of APP (C99). Re-probe of the blots with caspase-cleaved APP antibody detected two bands running at approximately 78 kDa, which were not altered under apoptotic conditions, and a time-dependent increase in the appearance of a proteolytic fragment of APP in lysates from apoptotic cells. Analysis of lysates from H4-APP cells after one, three, and six hours of treatments with CPT showed similar results on a 15% tris-glycine gel (Figure 2B, top and second panel). Reprobe of these blots with the caspase-cleaved APP antibody showed that two bands, which migrate between 25 to 37 kDa, are detected by this antibody (Figure 2B, third panel). This suggests that at least one of these bands may be similar to that detected by 6E10 antibodies. Quantification of the APP fragment (lower band) from three independent experiments showed a significant increase in APP cleavage under apoptotic conditions (Figure 2C). Since this antibody was more effective in detecting the fragments of interest, we decided to use it in further studies analyzing changes in fragment formation under different conditions. In order to determine whether these fragments are formed by caspase cleavage of APP, H4-APP cells were pretreated with either the group II caspase inhibitor Z-DEVD-FMK or the broad-spectrum caspase inhibitor Z-VAD-FMK for one hour, and then treated with or without CPT for six hours. Flow cytometric analysis of propidium iodide stained cells revealed that caspase inhibitors protected the cells against CPT-induced apoptosis (Figure 2D). Importantly, western blot analysis of the cell lysates revealed a significant reduction in CPT-induced generation of the novel fragments in the presence of Z-DEVD-FMK and more significant inhibition with Z-VAD-FMK (Figure 2E and Figure S1), suggesting that these fragments are generated in a caspase-dependent manner. The bar graph in Figure 2F shows the quantification of the cleaved fragments in the presence and absence of caspase inhibitors. Note that given the magnitude of these changes, a logarithmic scale was used.

### Activation of caspase-3 and -7 are associated with the formation of the observed fragments

**Figure 3:**
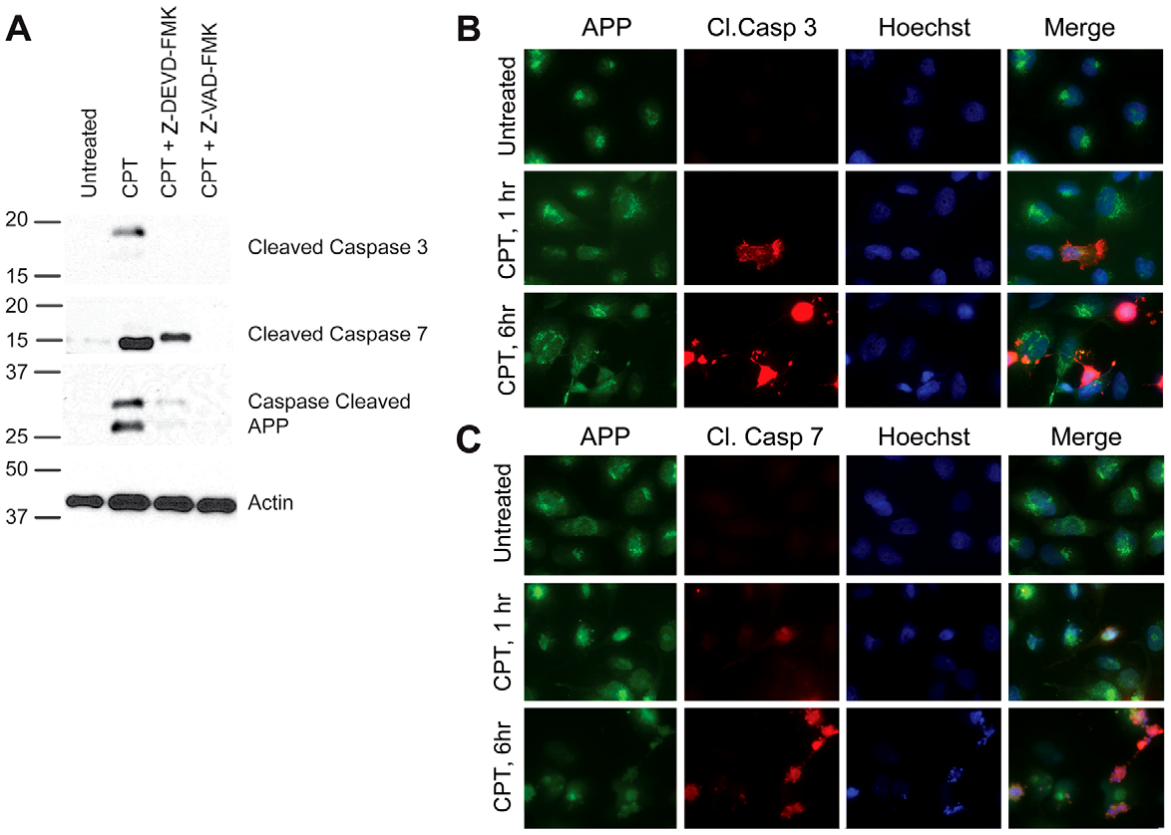
Activation of caspase-3 and -7 is associated with APP proteolysis in apoptotic cells. (A) H4-APP cells were treated with CPT in the presence and absence of caspase inhibitors for six hours and analyzed by western blot using antibodies directed against cleaved caspase-3 or cleaved caspase-7. Cleavage of caspase-3 (topmost panel) and caspase-7 (second panel) was observed in cells treated with CPT. Cleavage of these caspases was associated with an induction in the formation of the fragments detected by caspase-cleaved APP antibody (third panel). Blots were probed for β-actin as a loading control (bottom panel). Immunocytochemical analysis of H4-APP cells also showed significant induction in cleaved caspase-3 (B) and cleaved caspase-7 (C) after one and six hours of exposure to CPT. Cells were co-immunostained with 6E10 antibody (green) to show colocalization of both active caspase-3 and active caspase-7 (red) with APP (Magnification: 63X). Cells showing increased levels of cleaved caspases also showed a reduction in the level of APP signal intensity, consistent with the decrease in full length APP observed by western blot in Figure 2A and 2B.

In order to determine whether caspase-3 and/or caspase-7 may be responsible for the cleavage of APP in our model, we assessed levels and localization of cleaved, active caspases in cells undergoing apoptosis in response to CPT. Both cleaved caspase-3 and cleaved caspase-7 were significantly induced following a six hour treatment with CPT (Figure 3A). We observed a slight mobility shift in cleaved caspase-7 in cells treated with Z-DEVD-FMK, the reason for which is unclear at this point (Figure 3A, second panel). When compared to cleavage of caspase-3, cleavage of caspase-7 seemed to correlate more closely with the formation of these novel fragments (Figure 3A, third panel), suggesting that activation of caspase-7 may be associated with proteolytic cleavage of APP and generation of these fragments. Induction of cleaved caspase-3 and cleaved caspase-7 were also evident by immunocytochemistry after CPT treatment (Figure 3B and 3C, respectively). Both cleaved caspase-3 and cleaved caspase-7 co-localized with 6E10 staining in H4-APP cells, suggesting that this close proximity of APP with active caspases in apoptotic cells may facilitate APP proteolysis. The levels of APP in caspase-positive cells were lower compared to non-apoptotic cells.

### Apoptosis in neurons is associated with caspase activation and APP proteolysis

**Figure 4:**
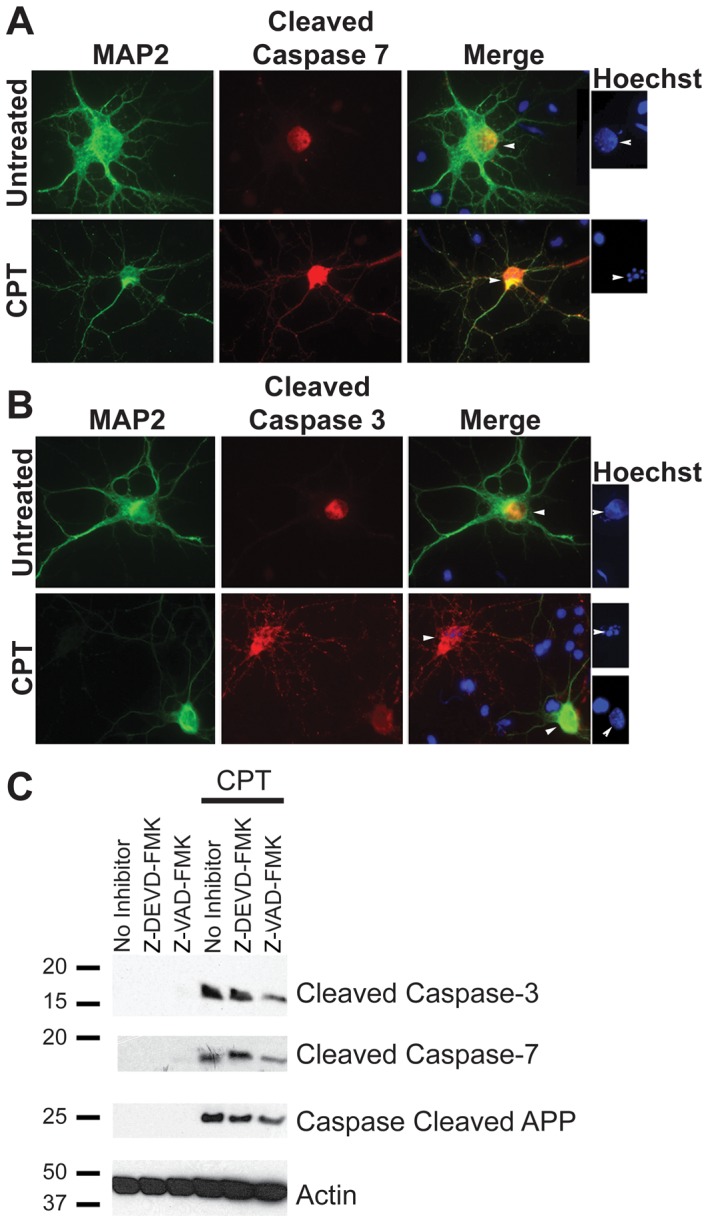
Apoptosis is associated with induction of cleaved caspase-3 and cleaved caspase-7 in primary cortical neurons. Primary neurons were cultured from E18 embryonic rat cortices for seven days, followed by treatment with 10 µM CPT to induce apoptosis. A and B show immunocytochemistry with MAP2 as a neuronal marker (green) and cleaved caspase-7 (A) or cleaved caspase-3 (B) antibodies (red) (Magnification: 63X). Hoechst staining, shown to the right of panels A and B, shows condensed or fragmented nuclei (indicative of apoptosis) in cells positive for cleaved caspases, whereas MAP2 positive neurons in the untreated samples show intact nuclei. (C) Western blot analysis of the lysates from neurons treated with CPT for 12 hours showed a significant induction in cleaved caspase-3 (top panel) and cleaved caspase-7 (second panel) levels. Activation of caspases was partially attenuated in the presence of 10 µM Z-DEVD-FMK and Z-VAD-FMK. Analysis of the lysates with an antibody generated against caspase-cleaved APP detected a single band of ∼25 kDa size. The level of this fragment was attenuated slightly in the presence of caspase inhibitors (Figure 4C, third panel). A reprobe of this blot using an antibody against β-Actin was performed as a control for protein loading (Figure 4C, bottom panel).

Caspase-3 and caspase-7 up-regulation and activation have been observed in AD brains [16]. Although caspase-3 activation has been associated with apoptosis in neurons [33], the role of caspase-7 in this process has not been established. To assess whether caspase-7 activation occurs in neurons undergoing apoptosis, we analyzed rat cortical neurons treated with CPT. Neurons were treated with CPT for 12 hours and immunostained using antibodies against cleaved caspase-3 or -7. MAP2 was used as a neuronal marker. Analysis of the cells under a fluorescent microscope showed a significant induction in the levels of cleaved caspase-3 and cleaved caspase-7 in neurons undergoing apoptosis (Figure 4A and 4B). In cells that showed substantial caspase activation, staining with the neuronal marker MAP2 was diminished, though MAP2 staining in the distal neurites (not shown) persisted. Together with cell morphology, this suggests that despite decreased MAP2 immunoreactivity these cells are indeed dying neurons. Western blot analysis of neuronal lysates treated with CPT for 12 hours also showed induction of both cleaved caspase-3 and cleaved caspase-7 in CPT treated cells (Figure 4C). This could be partially inhibited by the group II caspase inhibitor Z-DEVD-FMK and the pan-caspase inhibitor Z-VAD-FMK. Analysis of the cell lysate with caspase-cleaved APP antibody revealed the formation of an ∼25 kDa fragment under apoptotic conditions, the levels of which were attenuated in the presence of caspase inhibitors. This suggests that both caspase-3 and caspase-7 are present in neurons and are activated under apoptotic conditions.

### shRNA-mediated knockdown of caspases leads to a decrease in APP fragment formation

**Figure 5:**
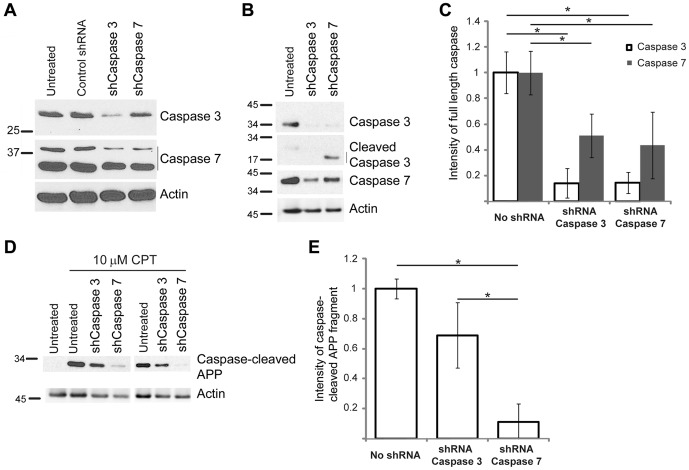
Down-regulation of caspase-3 and caspase-7 reduces the generation of ∼25–35 kDa caspase-cleaved APP fragments in CPT treated H4-APP cells. (A) H4-APP cells were transiently transfected with shRNA to caspase-3 or shRNA to caspase-7 and the down-regulation of the respective caspases analyzed after 48 hours using antibodies against caspase-3 (top panel) and caspase-7 (second panel). The lower panel in (A) shows an actin probe of the blot to show protein loading. (B) H4-APP cells stably transfected with shRNA to caspase-3 or caspase-7 were analyzed using the respective caspase antibodies. Reprobing of the blot with β-actin antibody shows protein levels on the blot (bottom panel). (C) Quantification of the levels of caspase-3 and caspase-7 in stably transfected cells from three independent experiments (as represented in B), normalized to β-actin. An approximately 86% decrease in full-length caspase-3 levels were observed in cells transfected with shRNA to caspase-3 or shRNA to caspase-7. Similarly, an approximately 49% decrease in caspase-3 levels was observed with shRNA to caspase-3, and a 56% decrease in caspase-7 levels was observed with shRNA to caspase-7. Induction of cleaved caspase-3 was observed with shRNA to caspase-7, but no induction of cleaved caspase-7 was observed with shRNA to caspase-3. (D) Western blot analysis of the lysates with antibody to caspase-cleaved APP showed a reduction in the levels of the cleaved fragment in the shRNA transfected cells after three hours of treatment with 10 µM CPT. The figure shows data from two experiments treated with CPT (E) Quantification of data from three independent experiments (representative figure shown in panel D), normalized to actin, shows a 31% reduction in caspase-cleaved APP with shRNA to caspase-3 and a 90% reduction in cells transfected with shRNA to caspase-7. Asterisks indicate significant differences, p<0.05.

The similarity in substrate specificity and lack of specific inhibitors capable of discriminating between group II caspases make it challenging to determine whether a specific proteolytic event is attributable to caspase-3, caspase-7, or both. Although it is classically difficult, we attempted to down-regulate each specific caspase using shRNA to caspase-3 and caspase-7 in order to examine whether down-regulation of these caspases have any effect on cleavage of APP to generate the fragments we have observed. Sets of four shRNA clones each to caspase-3 and caspase-7 were obtained from OriGene (Rockville, MD) and were transiently transfected into H4-APP cells using Turbofectin 8.0 (OriGene) according to the manufacturer's protocol. Initial studies showed that maximum down-regulation was obtained with clones one and four of each shRNA set, and we decided to use clone four of each shRNA set in our studies. Western blot analysis showed no down-regulation with a control shRNA (Figure 5A, lane 2 top and middle panel), while cells transiently transfected with shRNA to caspase-7 showed down-regulation predominately of caspase-7 (Figure 5A, lane 4). Cells transiently transfected with shRNA to caspase-3 showed down-regulation of both caspase-3 and caspase-7, indicating that the shRNA to caspases-3 was not specific for this caspase (Figure 5A, lane 3). Cells transfected with shRNA to caspase-7 showed down-regulation of two bands around 28–38 kDa on the western blots, which could be splice variants of procaspase-7 [34]. From the transient transfection experiments, we conclude that: (1) The effect of shRNA transfection is sequence dependent, as the control shRNA showed no effects, (2) shRNA to caspase-7 down-regulates caspase-7, and (3) the shRNA to caspase-3 used down-regulates both caspase-3 and caspase-7.

We also performed stable transfections of these same shRNAs into H4-APP cells (Figure 5B and 5C) and detected a significant reduction in full length caspase-3 and -7 with both shRNA clones (Figure 5B, top panel and third panel). Interestingly, in addition to inhibiting the expression of caspase-7, cells stably transfected with shRNA to caspase-7 showed an increase in cleaved caspase-3 (Figure 5B, second panel). Cells transfected with shRNA to caspase-3 did not show any up-regulation of cleaved caspase-7. This is not surprising as caspase-3, which is involved in the cleavage of caspase-7, was downregulated in these cells. Figure 5B shows a representative blot from one of three independent experiments; the results from these experiments were quantified and are presented in Figure 5C. Data from the stable shRNA transfection studies suggest that: (1) Similar to transient transfections, shRNA to caspase-3 resulted in down-regulation of both caspase-3 and caspase-7, (2) as with transient transfection, shRNA to caspase-7 was specific for caspase-7, and (3) stable knockdown of caspase-7 leads to an increase in cleavage of caspase-3 to the active form.

We next examined APP fragment formation in stably transfected cells treated with or without CPT by western blot analysis using the caspase-cleaved APP antibody (Figures 5D and 5E). Cells stably transfected with shRNA to caspase-3 showed a partial reduction in the levels of the cleaved fragments, while shRNA to caspase-7, which downregulated caspase-7 and induced cleavage of caspase-3, resulted in a substantial decrease in the levels of the fragments. This suggests that, although both caspase-3 and -7 may be capable of cleaving APP, caspase-7 is more directly associated with the formation of the fragments, as substantial down-regulation of these fragments was observed with shRNA to caspase-7, even in the presence of increased levels of cleaved caspase-3.

### Bioinformatic analysis of APP sequence identifies potential group II caspase cleavage site at DEVD*E_563_

**Figure 6:**
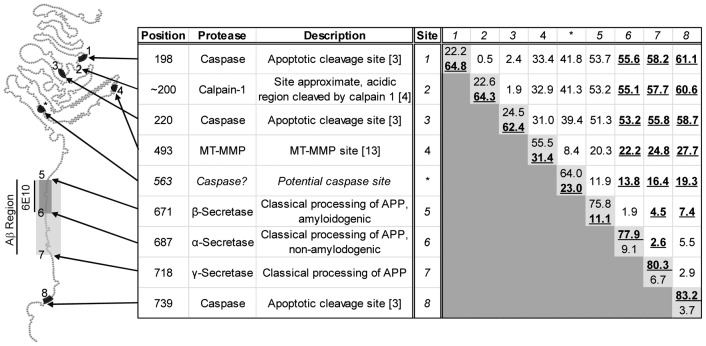
Bioinformatic prediction of potential proteolytic sites involved in generation of the novel APP fragments. The figure shows a schematic depicting known proteolytic sites on APP, and table identifying these various sites on APP_770_ isoform. Each site is assigned a number starting from the N-terminus, with a putative caspase cleavage site (DEVD* E_563_) identified by a support vector machine-based caspase substrate predictive model identified using an asterisk. The right side of the table is a grid illustrating the predicted molecular weights of fragments generated by cleavage at one or two of the sites identified. Each cell contains the predicted molecular weight for a fragment generated by cleavages at the site identified in the left side of the table and by number in the top row of the grid. The light gray cells on the diagonal, those where both row and column represent the same site, indicate cleavage at a single site with predicted molecular weight of the N-terminal and C-terminal fragments listed, respectively. The white cells show the molecular weight of the internal fragment generated by cleavage at two different sites. The molecular weights of fragments containing the 6E10 epitope (Aβ_1–16_) are bolded and underlined. Note that sites in the near extracellular domain, specifically the putative caspase site and the MT-MMP site, could potentially generate fragments similar in size and immunoreactivity to the fragments we observed.

The novel APP fragments observed in lysates from cells undergoing apoptosis immunoreacted with 6E10 and the C-terminal caspase cleavage site-specific antibodies. The only fragment generated by secretase cleavage of APP that would immunoreact with both of these antibodies would be the C99 fragment that is truncated by cleavage at VEVD*A_740_. Such a fragment would have a predicted molecular weight of ∼8 kDa. As this is considerably smaller than the molecular weight of the fragments generated under apoptotic conditions, we scanned APP for other known proteolytic sites, such as those cleaved by calpains, caspases, and MT-MMPs, to determine whether APP fragments with molecular weights similar to those we observed could be generated by proteases other than the secretases. Figure 6 shows a ball-and-chain model of APP with various proteolytic sites numbered. The grid towards the right of Figure 6 shows the predicted molecular weight of fragments generated by cleavage at a single site (light gray boxes, showing MW of N-terminal and C-terminal fragments respectively), or the internal fragment generated by cleavage at two sites identified by the intersection of a row and column. For example, cleavage at the β-secretase site (row 5) and the γ-secretase site (column 7) generates a fragment with a predicted molecular weight of ∼4.5 kDa, Aβ. These estimated molecular weights are based on the amino acid structure at neutral pH with no post-translational modifications and are calculated using the APP_770_ isoform. For fragments containing the 6E10 epitope, the molecular weight is bolded and underlined.

Since we suspected that the proteolytic processing may be caspase-dependent based upon the response to inhibitors and shRNA, we also utilized a support vector machine (SVM)-based predictive model to detect potential caspase cleavage sites in APP [35]. We identified a potential group II caspase site, DEVD*E_563_, approximately 25 kDa from the C-terminal end of APP, indicated by the asterisk in Figure 6. Based upon the predicted molecular weight of the fragments identified by bioinformatic analysis, we suspect that the fragments observed in this study are generated by cleavage of APP at either this novel caspase site or the site recognized by MT-MMPs in the extracellular domain and the caspase domain at VEVD*A_740_. Interestingly, cleavage in this manner would not preclude amyloidogenic or nonamyloidogenic processing of APP. It would, however, prevent the generation of the α-secretase-cleaved N-terminal fragment of APP, sAPPα, which has neuroprotective and anti-apoptotic properties. Given these protective properties, depletion of this fragment may have deleterious effects on cell survival.

### CPT treatment is associated with decreased production of sAPPα and Aβ

**Figure 7:**
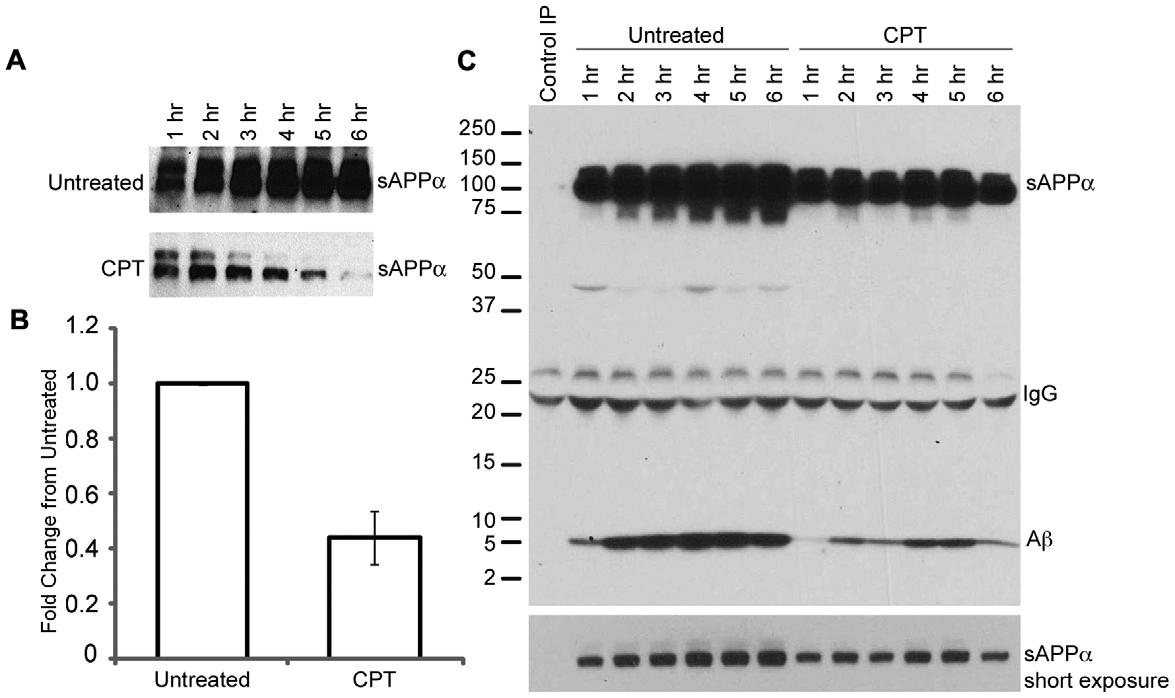
CPT treatment is associated with decreased production of sAPPα and Aβ. A) H4-APP cells were treated with or without 10 βM CPT and tissue culture supernatants were collected at every hour for six hours. The supernatants were boiled with Laemmli sample buffer and analyzed by PAGE and western blot using 6E10 antibodies. B) Quantification of data from untreated and CPT treated cells after six hours of treatment. C) Tissue culture supernatant from H4-APP cells treated with or without CPT for one to six hours was immunoprecipitated using 6E10 antibody and western blotted using the same antibody. CPT treatment resulted in a decrease in the levels of sAPPα as well as Aβ in the tissue culture supernatant (representative of two independent experiments).

In order to determine whether sAPPα production was indeed reduced in cells exposed to CPT, we next measured the secretion of sAPPα in apoptotic and non-apoptotic cells. H4-APP cells were treated with 10 µM CPT or left untreated and media was collected every hour for six hours. Media samples were analyzed by western blot using 6E10 antibody to determine the levels of sAPPα. A significant reduction in the levels of sAPPα was observed in the tissue culture supernatant from cells treated with CPT as compared to the levels in media from untreated cells, indicating altered processing of APP (Figure 7A and 7B). This decrease was sometimes evident as early as one hour after treatment with CPT, when the apoptosis was not yet detectable, suggesting caspase activation and APP proteolysis may occur prior to apoptosis. While cleavage in the near extracellular domain would preclude generation of sAPPα, the resulting C-terminal fragment would still contain the entire Aβ domain. In order to assess the effect of apoptosis on Aβ secretion, we treated H4-APP cells with or without CPT for one to six hours and examined the tissue culture supernatants by immunoprecipitation and western blot analysis using 6E10 antibody. Both secreted APP and Aβ levels were reduced in the tissue culture supernatants from CPT treated cells compared to the untreated controls, and this reduction was visible at all the time points examined (Figure 7C). Thus, in our experimental system apoptosis was not associated with increased secretion of Aβ, but it was associated with a decrease in the level of sAPPα. Since sAPPα has been implicated in various cellular functions including neuroprotection, neuronal excitability, and synaptic plasticity, our findings suggest that circumstances that result in altered processing of APP may enhance neurodegeneration through depletion of sAPPα in the cellular microenvironment, even when the levels of Aβ are low.

## Discussion

Here we report that under apoptotic conditions, APP is processed alternatively to generate fragments that are detected by antibodies directed towards Aβ region and C-terminal caspase cleavage site on APP. Based upon the size and immunoreactivity we conclude that the generation of these fragments involves cleavage within the ectodomain of APP, beyond the β-secretase site. Experiments with small peptide inhibitors of caspases and shRNA to caspases-3 and -7 suggest that these fragments are generated by a mechanism involving group-II caspases, and preferentially involving caspase-7. Based upon fragment size, immunoreactivity, and bioinformatic analysis of the APP sequence, we hypothesize that these fragments may be generated by cleavage of APP within the near ectodomain, potentially by MT-MMPs or caspases, and within the C-terminal domain by caspases. Cleavage within the near ectodomain prevents the production of sAPPα, which is neuroprotective and anti-apoptotic.

We used H4 neuroglioma cells overexpressing APP as a model system and induced apoptosis using the DNA damaging agent CPT. Although the levels of apoptosis is higher in our *in vitro* system as compared to the human condition, a near-homogeneous population of APP expressing apoptotic cells allows us to study changes in potentially intermediate proteolytic fragments of APP generated during apoptosis. In the human condition, even small changes in APP processing, over the decades-long disease process, may have significant impact on pathology development. Changes in sAPPα are of particular interest as sAPPα has been implicated in protecting neurons and synapses from apoptosis [36]. Our studies suggest that changes in APP processing associated with apoptosis could lead to decreased levels of sAPPα in the local microenvironment, making cells in that environment more prone to degeneration. Whether the altered processing of APP described here occurs *in vivo* in human AD brains needs to be established. However, analysis of brain extracts from mice overexpressing human APP [37]–[39] and human AD patients [40] have shown generation of similarly sized proteolytic products detected by 6E10 antibody. These APP metabolites are often interpreted as oligomeric Aβ, though they may in fact be proteolytic fragments of APP that contain the 6E10 epitope. Further, proteomic analysis of cerebrospinal fluid from AD patients has resulted in the identification of 11 short peptides that vary in N-terminal start site but end just before the α-secretase cleavage site. The N-termini of these fragments are formed by cleavages that occur in the near extracellular domain [41]. The precise origin of these fragments and the proteases that generate them are not clear, but their existence suggests that there is active proteolysis in the ectodomain region of APP, beyond the β-secretase site, in human AD brains. Taken together these data, as well as the results presented here, suggest that APP undergoes altered processing under degenerative and apoptotic conditions, and further detailed analysis of APP proteolysis is warranted to identify hitherto unknown intermediates as well as the importance of these cleavages in pathology development.

Apoptosis in neurons has been shown to be associated with caspase-3-mediated cleavage of APP at the C-terminus [42]. This cleavage of APP generates a 31 amino acid fragment from the C-terminus of APP, C31, which has been shown to be neurotoxic [43]. The effect of caspase cleavage on secretase processing of APP and Aβ production is not clearly understood. Co-localization of active caspase-3 with amyloid plaques and with caspase-cleaved APP has been observed in AD brain, which led to the hypothesis that caspase activation may lead to enhanced Aβ generation and plaque formation [6], [44]. However, this correlation does not necessarily indicate that caspase cleavage of APP increases Aβ generation, especially given that truncation of APP at the C-terminus removes the endocytosis signal from APP and affects its internalization. If the internalization of APP is reduced by caspase-mediated truncation this may in fact decrease Aβ production as endocytosis is required for cleavage of APP via the amyloidogenic pathway [45]. Our results support this hypothesis and show that apoptosis is associated with a decrease in generation of Aβ and sAPPα, as well as an increase in the formation of alternatively processed APP fragments.

Further, our data also suggest that in addition to cleavage by caspase-3, APP can be cleaved by caspase-7, and that caspase-7 may cleave APP at a novel site in the ectodomain. Such a cleavage would generate a C-terminal fragment of ∼25 kDa. This cleavage would likely occur after endocytosis of APP, within a luminal space, as DEVD*E_563_ lies in the extracellular domain of APP. Interestingly, caspase-7, but not caspase-3, has been shown to translocate to an endosomal-lysosomal compartment under inflammatory and apoptotic conditions [46]. This would allow caspase-7, but not caspase-3, access to DEVD*E_563_, explaining the apparent preference for caspase-7 over caspase-3 observed in our studies. Once APP is cleaved at this novel site, the fragment generated could undergo further proteolysis. We observed a decrease in the levels of secreted Aβ and C99 fragments in cells undergoing apoptosis, which is paralleled by a decrease in full-length APP as well as sAPPα. While changes in the levels of these fragments may be indicative of decreased expression of APP [47], the concomitant increase in the ∼25–35 kDa fragments observed here suggests that these changes are at least partially due to altered proteolysis. Formation of these fragments precludes the production of sAPPα, and the fragments themselves could be further processed by secretases and caspases, including cleavage by caspase-3 to generate the neurotoxic fragment C31. Further analysis of ectodomain cleavage of APP and the functions of the fragments generated is certainly warranted, as any alterations in APP processing may have important implications for pathology development in AD.

Studies in individuals with an increased risk for developing AD (those with a Clinical Dementia Rating of 0.5) show up-regulation of caspase-1 and caspase-7 in the entorhinal cortex [16]. Caspase-1, also known as interleukin converting enzyme, is associated with neuroinflammation and has been shown to cleave and activate caspase-7 *in vitro* [48]. The up-regulation of both of these caspases early in the disease process suggests that neuroinflammation may induce activation of caspase-7 and alternative processing of APP. Up-regulation of caspase-1 and caspase-7, but not TUNEL-positive cells, have been reported in the brains of patients with a Clinical Dementia Rating of 0.5. Patients with more advanced dementia (Clinical Dementia Rating of 5) show up-regulation of other caspases, including caspase-3, and a massive increase in TUNEL-positive cells [16]. This indicates that, despite its traditional role as an executioner caspase, caspase-7 may actually be acting early in the disease process, prior to the onset of apoptosis, and may play a physiological role in neurons outside of the apoptotic pathway. This is supported by our shRNA experiments in which caspase-3 cleavage was observed in cells stably transfected with shRNA to caspase-7, even under non-apoptotic conditions (Figure 5). The compensatory up-regulation of cleaved caspase-3, the closest homologue to caspase-7, under non-apoptotic conditions suggests an important physiological role for caspase-7 outside of apoptosis. While the role of caspase-3 in APP cleavage is beginning to be well understood, the role of caspase-7 in neuronal function, as well as caspase-7 mediated cleavage of APP, warrants further investigation.

The notion that caspase activation has a physiological role outside of apoptosis is not new. Caspases are thought to play an important role in modulating synaptic plasticity and long term memory [49]–[52]. APP and its proteolytic fragments have also been implicated in synaptic plasticity and neurite outgrowth [for review, see 53]. sAPPα has been shown to have substantial trophic effects on synaptic plasticity [54]. At low concentrations even Aβ appears to have a positive effect on synaptic plasticity [55], though at high concentrations Aβ aggregates and induces neurodegeneration [56]. The alternative APP processing events described in the studies presented here preclude the generation of the neurotrophic sAPPα fragment. This is confirmed by the observation that sAPPα levels are decreased under apoptotic conditions. They do not, however, preclude the formation of the neurotoxic Aβ and C31 fragments. It is possible that the fragments generated under apoptotic conditions are intermediate fragments of APP which may be further processed by secretases to generate Aβ and/or C31. The cleavage of APP that generates these fragments may, therefore, enhance neurodegeneration by decreasing the availability of sAPPα to provide neuroprotection while still allowing the production of toxic fragments. This suggests that neuroinflammation-dependent caspase-7 activation could shift APP into an alternative cleavage pathway that is potentially cytotoxic, and inhibition of this pathway may protect neurons against cytotoxicity.

## Conclusion

In conclusion, our studies suggest that APP is cleaved in the extracellular domain, beyond the β-secretase site, to generate fragments that contain the Aβ region and the intracellular domain of APP. Because proteolytic processing of APP has important implications for disease pathogenesis and synaptic plasticity, the molecular pathways governing the generation of these fragments, including the potential involvement of caspase-7 in APP proteolysis, warrant further investigation. An understanding of the role of these proteases in APP proteolysis as well as a thorough characterization of the functions and effects of the proteolytic fragments generated by these proteases are essential to our knowledge of how APP affects both AD pathogenesis and normal synaptic function. Due to the dogma that caspases are associated with the final stages of apoptotic cell death, the notion of caspase inhibition as a therapeutic approach in AD is sometimes regarded as “too little, too late”. However, the evidence presented here that caspase-7 is involved in APP proteolysis, as well as the previously reported observation that caspase-7 is up-regulated early in the clinical progression of dementia [16], suggests that caspase-specific inhibitors have the potential to modulate disease progression and neurodegeneration by preventing alternative processing of APP.

## Supporting Information

Figure S1 **Inhibition of APP cleavage by caspase inhibitors.** Treatment of H4 glioma cells with caspase inhibitors show inhibition of APP processing: H4 glioma cells were left untreated or treated with CPT for one, two, three or six hours or pre-treated for one hour with Z-DEVD-FMK or Z-VAD-FMK and treated with CPT for six hours and cell lysates were prepared and separated on a 15% tris-glycine gel and analyzed by western blot using 6E10 (A) and caspase cleaved APP (B & C) antibodies. 6E10 antibody (A) shows the appearance of the cleaved fragment of APP with a concomitant decrease in the levels of full length APP (top panel) after CPT treatment. The formation of the fragment is inhibited by treatment of the cells with Z-DEVD-FMK and Z-VAD-FMK, which was associated with slight increase in full length APP. Caspase-cleaved APP antibody shows a much stronger immunoreactivity to the proteolytic fragments (C, long exposure). The short exposure (B) from the caspase-cleaved APP blot shows inhibition of band formation by z-DEVD-FMK and Z-VAD-FMK, the long exposure shows that the inhibition was more efficient in cells treated with Z-VAD-FMK. Probing for β-actin (D) showed equal amount of protein loading on the gels.
(TIF)
