# Supplementary figures and images for: Altered Processing of Amyloid Precursor Protein in Cells Undergoing Apoptosis
Source: PLoS One. 2013 Feb 28;8(2):e57979. doi: 10.1371/journal.pone.0057979 (PMC3585261; doi:10.1371/journal.pone.0057979)

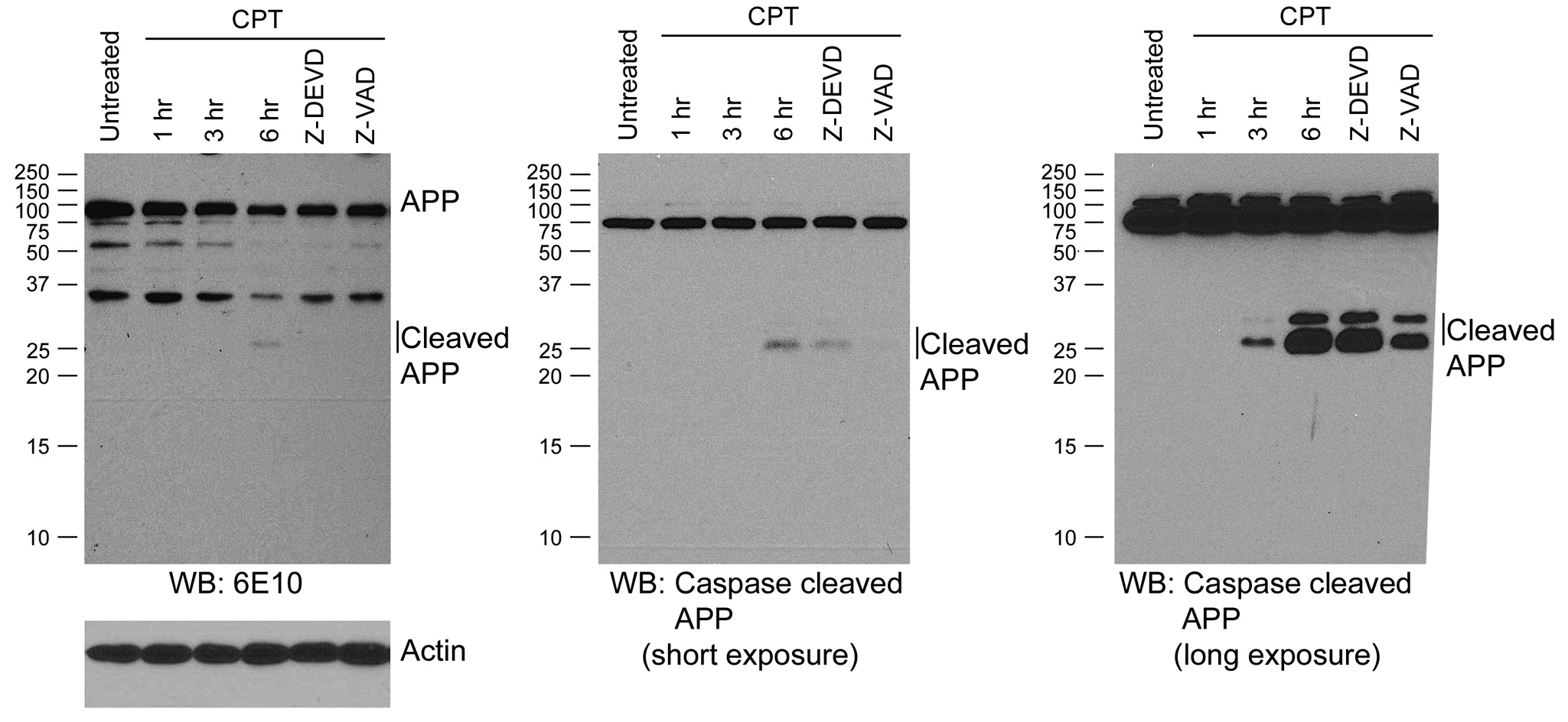

Supplement: Figure S1 — Inhibition of APP cleavage by caspase inhibitors. Treatment of H4 glioma cells with caspase inhibitors show inhibition of APP processing: H4 glioma cells were left untreated or treated with CPT for one, two, three or six hours or pre-treated for one hour with Z-DEVD-FMK or Z-VAD-FMK and treated with CPT for six hours and cell lysates were prepared and separated on a 15% tris-glycine gel and analyzed by western blot using 6E10 (A) and caspase cleaved APP (B & C) antibodies. 6E10 antibody (A) shows the appearance of the cleaved fragment of APP with a concomitant decrease in the levels of full length APP (top panel) after CPT treatment. The formation of the fragment is inhibited by treatment of the cells with Z-DEVD-FMK and Z-VAD-FMK, which was associated with slight increase in full length APP. Caspase-cleaved APP antibody shows a much stronger immunoreactivity to the proteolytic fragments (C, long exposure). The short exposure (B) from the caspase-cleaved APP blot shows inhibition of band formation by z-DEVD-FMK and Z-VAD-FMK, the long exposure shows that the inhibition was more efficient in cells treated with Z-VAD-FMK. Probing for β-actin (D) showed equal amount of protein loading on the gels. (TIF) [file pone.0057979.s001.tif]
